# Rapid Resolution of Large Bowel Diarrhea after the Administration of a Combination of a High-Fiber Diet and a Probiotic Mixture in 30 Dogs

**DOI:** 10.3390/vetsci7010021

**Published:** 2020-02-10

**Authors:** Giacomo Rossi, Matteo Cerquetella, Alessandra Gavazza, Livio Galosi, Sara Berardi, Sara Mangiaterra, Subeide Mari, Jan S. Suchodolski, Jonathan A. Lidbury, Joerg M. Steiner, Graziano Pengo

**Affiliations:** 1 School of Biosciences and Veterinary Medicine, University of Camerino, Via Circonvallazione 93/95, 62024 Matelica (MC), Italy; giacomo.rossi@unicam.it (G.R.); matteo.cerquetella@unicam.it (M.C.); livio.galosi@unicam.it (L.G.); sara.berardi@unicam.it (S.B.); sara.mangiaterra@unicam.it (S.M.); subeide.mari@unicam.it (S.M.); 2 Gastrointestinal Laboratory, Department of Small Animal Clinical Sciences, Texas A&M University, College Station, TX 77843, USA; jsuchodolski@cvm.tamu.edu (J.S.S.); jlidbury@cvm.tamu.edu (J.A.L.); jsteiner@cvm.tamu.edu (J.M.S.); 3 St. Antonio Veterinary Clinic, S.S. 415 Paullese 6, 26020 Madignano (CR), Italy; graziano@cvsantantonio.eu

**Keywords:** colitis, food responsive diarrhea, gut homeostasis, high fiber, therapy

## Abstract

Canine fiber responsive diarrhea is a form of chronic colitis that improves clinically after adding fiber to the diet. In the present study, we investigated the effect of a combination of a high-fiber, highly digestible, hypoallergenic diet with a probiotic mixture in 30 dogs with chronic colitis that were unresponsive to various dietary and/or pharmacological interventions. Fecal scores, canine chronic enteropathy clinical activity index (CCECAI) scores, the dysbiosis index (DI), and histologic images of colonic biopsies were evaluated. At baseline (day 0; T0) and after 30 days of treatment (T1), all variables evaluated in our patients (i.e., fecal and CCECAI scores and histopathology) improved significantly at T1, with the exception of DI. However, there was a numerical shift from a state of dysbiosis to one of normobiosis. The combination of the diet and the probiotic used in the present study induced the resolution of clinical signs in a mean of 8.5 days (maximum 15 days) and did not necessitate any other treatments or the further addition of alimentary fiber.

## 1. Introduction

Food responsive diarrhea is defined by chronic diarrhea that improves clinically in response to switching the patient to a new and exclusive diet, such as a limited antigen diet, a hydrolyzed protein diet, an easily digestible diet, a high-fiber diet, or some other diet [1]. The response to dietary modification is also the current way to diagnose this condition, as no specific markers are available [1,2,3]. Fiber responsive large bowel diarrhea (FRLBD) is a chronic condition mainly affecting the colon, which improves by adding fiber to the diet [4]. Although not all aspects of FRLBD have been clearly defined, it is interesting to note that it is considered as a sub-group of another condition called chronic idiopathic large bowel diarrhea (the other sub-group is associated with stressors and also named irritable bowel syndrome), and that all of these conditions recognize the use of dietary fiber in their management [5,6,7]. Thus, the main therapeutic approach in dogs with FRLBD consists in the administration of fiber, such as psyllium, or in the use of commercially available high fiber diets [4]. 

Many recent papers have reported on the effects of probiotic administration in the management of chronic gastrointestinal disease in both dogs and cats [8,9,10,11,12]. Most of these studies demonstrated multiple beneficial effects that can be, for example, attributed to their ability to improve dysbiosis, reduce inflammatory cell infiltrates and/or inflammatory receptor expression, and modulate motility [10,11,12].

In the present study, we aimed to evaluate the efficacy and safety of the combination of a commercially available high-fiber diet with a highly concentrated probiotic mixture in managing patients with fiber responsive large bowel diarrhea. All patients included, chronically suffering from colitis, clinically improved/solved their conditions in a mean time of 8.5 days.

## 2. Materials and Methods

### 2.1. Inclusion Criteria for Dogs and Diagnostic Work-Up

After a complete diagnostic work-up, thirty dogs (that completed the study out of the 115 that initially enrolled) with chronic large bowel diarrhea, which responded to a dietary trial and were successively diagnosed with FRLBD, were included. All patients had spontaneous disease, and treatment consisted of commercially available products (see below). The collection and analysis of intestinal biopsies obtained endoscopically from dogs included in the study were performed for clinical purposes, using routine techniques, respecting National Laws on Studies Involving Animals. The enrolled dogs and their owners received written information on methods, according to previous studies [13], and all owners gave their written informed consent to participate in the study.

The median duration of clinical signs before diagnosis was 38 weeks (range: 12–59). At T0, all dogs had one or more signs of chronic colitis, such as watery diarrhea several times a day, urgency, presence of mucus and/or fresh blood in the stool, but also vomiting, decreased appetite or anorexia, abdominal pain, lethargy, flatulence, and/or weight loss. No dogs included in the study had been subjected to any corticosteroids or antibiotics for the previous month, while all were variably subjected to different dietary changes over the previous months. 

The standard diagnostic protocol for dogs with chronic enteropathy included blood work (e.g., complete blood count, serum chemistry profile with electrolytes, etc.), urinalysis, diagnostic imaging (i.e., abdominal ultrasound and radiographs), and fecal examination (i.e., parasitology including *Giardia*) [6,7]. Dogs were then subjected to colonoscopy at T0 and at T1 (after 30 days of therapy); 5 dogs also underwent upper gastrointestinal (GI) endoscopic examinations due to clinical signs suggesting upper GI tract disease. At least 8 samples were randomly collected in the absence of macroscopic lesions, while all altered mucosal sites (i.e., areas that showed micro erosions, hyperemic/edematous/hemorrhagic areas, or areas with irregular/infiltrated mucosa) were also sampled. All biopsy samples examined in this study were taken by two operators, using a large biopsy forceps (2.4 mm). In attempt to provide more information at least one of the eight samples was taken up to the Muscularis mucosae.

Characteristics and clinical findings in the dogs enrolled in the study are reported in Table 1.

### 2.2. Therapeutic Intervention

For each dog, the therapeutic intervention consisted of a diet change and the administration of a probiotic mixture for 30 days. The diet chosen was Intestinal Colitis Phase 1 (currently Intestinal Colon Phase 1; SANYpet SpA—Forza10, Bagnoli di Sopra (PD), Italy) and the probiotic mixture administered was the Slab51^®^ bacterial blend (*Lactobacillus acidophilus*, *L. plantarum*, *L. paracasei*, *L. helveticus*, *L. brevis*, *Streptococcus thermophilus*, and a mix of two *B. lactis*), available in 200 billion bacteria per sachet (SivoMixx^TM^—Ormendes S.A., Lugano, Switzerland). The probiotics dosage was established according to a previous study from the authors [14] and based on the animal body weight: dogs weighing between 5 and 10 kg received ½ sachet daily, and those weighing 10 kg or more received 1 sachet per day. The diet was mainly chosen because it contains hydrolyzed fish protein and for its high dietary fiber content (21%; raw fiber content: 7.8%). 

### 2.3. Fecal Score 

Fecal quality was assessed and recorded by owners for every defecation, by using a 5-point visual scale. The scoring system ranged from 1, represented by hard and dry feces, to 5, which was considered consistent with liquid diarrhea. A score of 2 represented a well-formed stool that was easily collectible and not too dry (optimal fecal score). The mean fecal scores (FS) [15] were recorded at T0 and T1. The mean fecal score at T0 was the average of the values recorded by the owners for the seven days prior to T0; the FS at T1 was the average of the values recorded by the owners during the last week of treatment. 

### 2.4. CCECAI Score

The severity of clinical disease (activity) at inclusion (T0) and after treatment (T1) was scored using the canine chronic enteropathy clinical activity index (CCECAI) [16]. 

### 2.5. Dysbiosis Index

A fecal sample taken from each dog at T0 and the last day of therapy (T1), was immediately stored at −80 °C, until microbiome analysis. The abundances of select bacterial taxa (i.e., *Blautia* spp., *Clostridium hiranonis*, *E. coli*, *Faecalibacterium* spp., *Fusobacterium* spp., *Streptococcus* spp. and *Turicibacter* spp.) as well as total bacteria were measured by quantitative real-time PCR (qPCR) assays and the dysbiosis index (DI) was calculated as previously reported [17].

### 2.6. Histopathology 

After enrollment (time point T0) and after 30 days of treatment (T1), multiple colonic mucosal biopsy specimens were obtained endoscopically from all enrolled dogs (n = 30). Twenty-five patients underwent only colonoscopy, while five patients had both upper and lower endoscopic examinations. Biopsy specimens were obtained directly from areas with mucosal alterations if present (i.e., areas of edema, reddening, mild increased granularity, and/or friability), as well as from areas of normal-appearing mucosa. Tissue samples were placed in 10% buffered formalin solution for histopathologic evaluation, then paraffin embedded, and serial 3-μm thick sections were prepared. Hematoxylin-and-eosin- (H and E) stained tissue sections of paraffin embedded endoscopic biopsies from the colon of each dog were evaluated for histopathologic lesions. A single pathologist, who was blinded regarding the patients’ history, clinical signs, or endoscopic findings, performed the histopathologic evaluation. A severity score was assigned for each dog, by using a standardized histologic grading system, based on the extent of architectural disruption and mucosal epithelial changes referring to what previously was proposed by the World Small Animaly Veterinary Association (WSAVA) for diagnosis of gastrointestinal inflammation [18]. The scoring system is reported in the caption of Table 2.

### 2.7. Statistical Analysis

Data normality was tested using the Shapiro-Wilk Test. The differences between T0 and T1 for fecal score, CCECAI, and histopathology scores were determined by a Wilcoxon test. The differences in the dysbiosis index between T0 and T1 were assessed using a Student’s *t*-test. The differences were considered statistically significant at *p* < 0.05. Statistical analysis was performed using a commercial software package (MedCalc, v.15.8, Mariakerke, Belgium).

## 3. Results

After 15 days from the beginning of the study, all owners of the dogs were reporting a normalization of the feces quality with a fecal score ranging from 1 to 3 (*p* < 0.0001); the minimum time to normalization of fecal quality was 4 days, with a mean of 8.5 days (Table 1). The GI transit time also improved as evidenced by the number of daily defecations that were no more than three, or in some cases two (data not shown). Interestingly, in all subjects, the urgency for defecation disappeared at T1. Additionally, at the same time point, no more mucus of fresh blood in stools were reported, a very important aspect in those patients already having a normal fecal score at T0. 

Similarly, the CCECAI score (Table 1) significantly improved from T0 to T1 (*p* < 0.0001) and the median value between the two time points, decreased from 8 to 2. 

**Figure 1:**
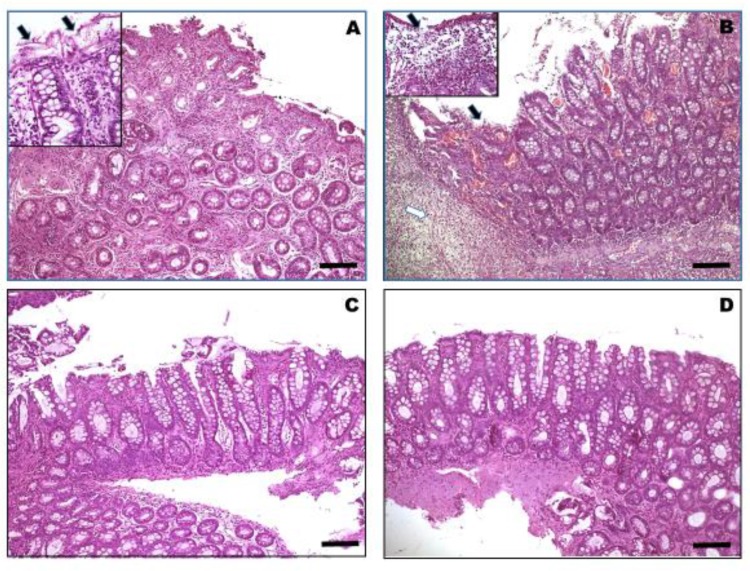
Tissue sections belonging to colonic mucosa biopsies of different dogs that were enrolled in the study and affected by chronic colitis, before (**A** and **B**) and after (**C** and **D**) the supplementation with high-fiber and probiotic diets. Histopathological features changed significantly before and after the treatment; colonic samples from treated dogs (C and D) showed a residual inflammatory infiltrate throughout the mucosal *lamina propria* and a substantial improvement in the epithelial layer is observed, with a correct proportion of goblet cells in the glandular and covering epithelium. Conversely, a histopathologic evaluation of colonic biopsies collected before changing the diet and starting the administration of the probiotic, reveals moderate to severe transmural infiltration with inflammatory cells associated with fibrosis (A) or with necrosis and areas of erosion/exulceration of colonic mucosa (B). (H and E stain, 10×. Scale bar = 250 µm).

The mean dysbiosis index (Figure 1) was positive at T0 (mean value 1.72), showing that dysbiosis was present. After treatment, the mean value for the DI was −0.80, indicating that normobiosis was reached, although this difference of the mean DI did not reach statistical significance (*p* = 0.079).

Finally, as reported in Table 2, histopathological features changed significantly, showing that although there was a residual inflammatory infiltrate present at T1, histological scores were significantly (*p* < 0.0001) reduced at T1 when compared to T0 in a large percentage of dogs (28/30; 93.3%) (See Figure 1). 

It is worth noting that, in the inserts, damaged epithelial areas represented by cellular swelling and ballooning (Figure 1A) associate to leukocytes transmigration and areas of lost epithelium (Figure 1B) and mucosal erosion due to morphological signs of apoptosis (arrows) and severe inflammatory cell infiltration (white arrow), which is mostly represented by lymphocytes and plasma cells. (H and E stain, 40×. Scale bar = 100 µm). 

Interestingly, before changing the diet and starting the administration of the probiotic, the histologic assessment of colonic biopsy samples showed moderate to severe transmural infiltration with inflammatory cells evidenced at different percentages of extension in biopsy samples and altering the normal architecture of the colon with an average score of 9.6. Conversely, a histopathologic evaluation of colonic biopsies collected after treatment revealed a variable recovery of the colonic architecture, with an average score of 3.3. Some colonic samples (five out of thirty) showed a partial restoration of the damaged epithelial layer (even if some areas of cellular swelling and ballooning with morphological signs of apoptosis persisted) in contrast with the extensive vacuolization/erosion of the colonic mucosa observed in pre-treated dogs. In addition, the infiltrate features shifted from slight to moderate with a patchy distribution, showing a lower number of inflammatory cells mostly represented by lymphocytes and plasma cells. Furthermore, five out of thirty samples showed evident and complete restoration of the epithelial cell layer, while in the rest of the samples the epithelial alterations of the mucosa affected over 40–50 % of the surface, lower than in most of the specimens taken before the treatment. Therefore, in 10 dogs out of 30 (33.3%) there was a partial to complete improvement of the mucosal epithelium of the colon. Similarly, the goblet cell depletion was also attenuated after diet change and probiotics administration, and the presence of mucin was evident with a lack of dilated crypts. Finally, the inflammatory infiltrate was reduced, being moderate with a patchy distribution.

## 4. Discussion

The main protein source of the diet used for this study was a hydrolyzed fish protein, while brown rice and rice were utilized as the carbohydrate source. Thus, this diet would be an attractive option for dogs with food intolerance. In addition, given the fiber content of the diet, this would also make it a good option for dogs suspected with food responsive diarrhea involving the colon. The high content of fiber in the diet comes from the addition of bio mannan-oligosaccharide (MOS), fructo-oligosaccharide (FOS), *Yucca schidigera*, and also from the seaweed *Ascophyllum nodosum*, resulting in a balanced supply of soluble and insoluble fibers [19]. *Ascophyllum nodosum* not only acts as a fiber source, but it is also “functional”, thanks to its remarkable concentration in fucoidans, a complex series of sulfated polysaccharides found widely in the cell walls of brown seaweeds and in particularly concentrated in *A. nodosum* [20]. The antioxidant, anti-inflammatory, anti-allergic, and anti-obesity properties of fucoidans have been described by Li and colleagues in 2008 [21], and these properties would suggest that fucoidans could be applied as part of functional foods for disease prevention and health promotion in mice and mammals [22]. Fiber in the diet is very important, as is the ability to modulate GI motility, but it is also associated with colonic mucosal hypertrophy, which is accompanied by increased colonic weight, suggesting a possible increase in the absorptive capacity [7,23]. 

Our idea to combine the above-mentioned highly digestible, hypoallergenic diet with the probiotic mixture was aimed at increasing the production of short chain fatty acids (SCFAs), through bacterial fermentation. SCFAs play many beneficial roles as they are necessary for restoring and/or maintaining a correct microbial balance (some fibers act as prebiotics) or, as is the case with butyrate, acting as an important energy source for colonocytes [7,24]. We were able to appreciate these effects not only based on clinical improvement, as evidenced by the significant improvement of the fecal score, but also based on the dysbiosis index and the scores for the histopathological evaluation. In the case of DI, it changed from a positive value at T0 to a negative value at T1, suggesting that the combination therapy was effective in restoring a healthy gut microbiome, eliminating the dysbiosis, although the difference of the DI between T0 and T1 was not significant. Histopathology showed a restoration of the epithelial lining in 30.3% and a reduction in the inflammatory infiltrate in 93.3% of the dogs enrolled in this study. It may be true that barrier disruption leads to increased stimulation through luminal antigens. In this regard, mucosal inflammation can be considered a self-perpetuating process in which the disruption of the epithelial layer plays a central role [25]. The combination of a high-fiber diet, probiotics, and a hydrolysed diet was able to reduce inflammatory cell infiltration in the colon of dogs, as was observed during the microscopic analysis. The reduction of mononuclear cell infiltration can account for the intestinal anti-inflammatory effect of this combination therapy given the important role attributed to these cells in the inflammatory process. Further evidence shows that the use of a probiotic, such as those containing *Lactobacillus*, *Bifidobacterium*, or *Enterococcus,* can decrease the levels of inflammatory cytokines [26]. Recently, Bonfili and colleagues found that the administration of the same probiotic formulation used in this study (Slab51^®^) significantly reduced the oxidative stress in a triple transgenic mouse model of Alzheimer’s disease, by inducing the Sirtuin-1-dependent mechanism [27]. In addition, in a rat model of colitis, a probiotic supplement was shown to counteract the depletion of colonic glutathione levels that took place in control colitic animals [28]. The anti-oxidative activity of the probiotic may play a role in the intestinal anti-inflammatory effect, as the oxidative insult is an important mechanism for tissue damage during chronic intestinal inflammation and is thus a common feature in human inflammatory bowel disease (IBD) [29], as well as in experimental models of rat colitis, including the 2,4,6-trinitrobenzene sulfonic acid (TNBS) [30] and the dextran sodium sulfate [31] models. The effect exerted by some probiotics could be due to their ability to release glutathione and the antioxidant dipeptide g-Glu-Cys [32], and to significantly reduce colonic TNFα production [32,33]. TNFα is a cytokine that plays a key role in intestinal inflammation, and different drugs capable of interfering with the activity of this mediator have been developed for IBD therapy in humans [34]. Recently, a decrease in oxidative stress and a reduction in gut mucosal TNFα levels were also demonstrated by feeding a murine model of endotoxemia a high-fiber diet [35]. These reports demonstrate the possible synergistic anti-inflammatory effects of a combination of a high-fiber diet and probiotic administration in the management of canine colitis. 

The main weakness of the present study was the absence of a control group, and the comparison between probiotics and probiotics plus fiber diet. However, the authors believe that this does not represent a significant limitation as our aim was not to demonstrate that fiber is a good therapeutic option in dogs with colitis, as the management of fiber-responsive large bowel diarrhea in dogs has been described in multiple publications [4,6,7,36]. We investigated how a combination diet and probiotic mixture supplementation could not only influence clinical progression but also have an impact on the gut microbiota, inflammatory infiltration, and tissue damage.

## 5. Conclusions

In the present study, we demonstrated that the combination of a high fiber diet plus a highly concentrated probiotic mixture is effective and safe for the management of dogs with fiber-responsive large bowel diarrhea, leading to the resolution of clinical sings in, on average, just over one week, and not necessitating any other treatments or the further addition of alimentary fiber (e.g., psyllium).

## Figures and Tables

**Table 1:** Breed, sex, and age of dogs enrolled in this study, as well as canine chronic enteropathy clinical activity index (CCECAI) scores and fecal scores at T0 and T1. The number of days required for stool normalization is also reported.

Dogs	Breed	Sex	Age	Days until Stool Normalization (Mean Value: 8.5)	Fecal Score *		CCECAI *	
					T0 (Median Value: 4)	T1 (Median Value: 2)	T0 (Median Value: 8)	T1 (Median Value: 2)
1	German Shepherd	M	2.5 years	6	4	2	9	2
2	Crossbreed	F	6 years	5	5	2	8	3
3	Border Collie	M	1.5 years	11	3	2	6	3
4	Boxer	M	2.5 years	10	5	3	9	3
5	Dobermann	F	2 years	7	4	1	9	1
6	Cocker Spaniel	F	10 years	9	3	2	7	0
7	Boston Terrier	M	6.5 years	11	4	3	8	2
8	Sussex Spaniel	M	8.5 years	15	5	3	10	4
9	Amstaff	M	12 years	9	4	2	8	5
10	Labrador Retriever	F	10 years	8	4	1	7	0
11	Boxer	M	1 year	7	5	3	7	3
12	German Shepherd	M	3.5 years	4	5	3	10	4
13	Crossbreed	M	11 years	5	4	1	8	1
14	German Shepherd Crossbreed	F	12 years	5	4	2	4	0
15	Magyar Vizsla	M	6 years	8	5	2	5	2
16	Wire-haired Dachshund	F	7.5 years	11	3	2	8	2
17	Crossbreed	M	3 years	9	3	1	4	1
18	Poodle	M	3 years	8	4	3	8	4
19	Boxer	M	2 years	13	5	3	8	3
20	Bernese Mountain Dog	M	< 1 year	10	5	2	9	0
21	Yorkshire Terrier	F	7.5 years	5	4	2	6	3
22	German Shepherd	M	1 year	8	4	2	9	4
23	Dobermann	F	< 1 year	10	5	3	11	5
24	Pinscher	M	2 years	4	4	2	8	2
25	Jack Russel Terrier	M	11 years	11	5	3	9	3
26	Rhodesian Ridgback	F	4 years	5	4	2	9	0
27	Italian Greyhound	M	< 1 year	14	3	3	7	0
28	Akita Inu	F	3 years	6	3	2	8	4
29	French Bulldog	M	2 years	15	2	2	4	2
30	English Setter	M	3 years	7	4	2	4	0

* (T0 vs T1) *p* < 0.0001.

**Table 2:** Histopathology scores for colonic biopsies calculated from all histopathologic variables considered. * (T0 vs T1) *p* < 0.0001.

Patient Number	T0 *		T1 *	
	Score by Variable ^§^	Total	Score by Variable ^§^	Total
1	3 + 2 + 3 + 4	**12**	1 + 1 + 0 + 1	**3**
2	2 + 2 + 2 + 3	**9**	1 + 1 + 1 + 1	**4**
3	2 + 2 + 1 + 2	**7**	1 + 1 + 0 + 1	**3**
4	3 + 3 + 4 + 4	**14**	1 + 2 + 0 + 1	**4**
5	3 + 2 + 4 + 4	**13**	1 + 1 + 0 + 3	**5**
6	2 + 2 + 2 + 2	**8**	2 + 1 + 2 + 2	**7**
7	2 + 2 + 2 + 3	**9**	1 + 1 + 0 + 3	**5**
8	3 + 3 + 4 + 4	**14**	1 + 1 + 0 + 1	**3**
9	2 + 3 + 3 + 3	**11**	1 + 1 + 1 + 1	**4**
10	3 + 2 + 4 + 4	**13**	1 + 1 + 0 + 3	**5**
11	3 + 3 + 4 + 4	**14**	1 + 1 + 0 + 1	**3**
12	3 + 2 + 4 + 4	**13**	1 + 1 + 0 + 3	**5**
13	2 + 2 + 2 + 3	**9**	1 + 1 + 0 + 1	**3**
14	2 + 2 + 2 + 2	**8**	2 + 2 + 2 + 3	**9**
15	1 + 1 + 1 + 1	**4**	1 + 1 + 1 + 1	**4**
16	2 + 2 + 2 + 2	**8**	0 + 0 + 0 + 0	**0**
17	1 + 1 + 0 + 1	**3**	0 + 0 + 0 + 0	**0**
18	2 + 2 + 2 + 3	**9**	0 + 0 + 0 + 0	**0**
19	3 + 2 + 4 + 4	**13**	1 + 1 + 0 + 0	**2**
20	3 + 3 + 4 + 4	**14**	1 + 1 + 0 + 1	**3**
21	2 + 2 + 2 + 3	**9**	1 + 1 + 0 + 1	**3**
22	3 + 2 + 3 + 4	**12**	1 + 1 + 1 + 1	**4**
23	3 + 3 + 4 + 4	**14**	1 + 1 + 0 + 1	**3**
24	2 + 2 + 1 + 2	**7**	1 + 1 + 1 + 1	**4**
25	3 + 2 + 3 + 3	**11**	1 + 1 + 0 + 1	**3**
26	3 + 2 + 3 + 4	**12**	1 + 1 + 1 + 1	**4**
27	1 + 1 + 1 + 1	**4**	0 + 0 + 0 + 0	**0**
28	3 + 2 + 2 + 2	**9**	1 + 1 + 0 + 1	**3**
29	1 + 1 + 0 + 1	**3**	0 + 0 + 0 + 0	**0**
30	1 + 1 + 1 + 1	**4**	1 + 1 + 0 + 0	**2**
**Median value 9**			**Median value 3**	

^§^ Colonic pathology was scored using a previously adapted scoring system (modified by Day et al., 2008), the variables considered were: the severity of the inflammatory infiltration (scored as: 0 (none), 1 (mild), 2 (moderate), and 3 (severe)); the extent of the inflammation (scored from 0 (none), 1 (mucosal inflammation), 2 (mucosal and submucosal inflammation), and 3 (transmural inflammation); Crypt Damage (scored as 0 (none), 1 1/3rd of crypts damaged, 2 2/3 or crypts damaged, 3 crypts lost but surface epithelium present, and 4 (crypts and surface epithelium lost); the extent of involvement (scored as 0: 0% involvement, 1: 1–25% involvement, 2: 26–50% involvement, 3: 51–75% involvement, and 4: 76–100% involvement). The maximum score of this scoring system is 14 points.
